# A three dimensional visualisation approach to protein heavy-atom structure reconstruction

**DOI:** 10.1186/s12900-014-0027-8

**Published:** 2014-12-31

**Authors:** Xubiao Peng, Alireza Chenani, Shuangwei Hu, Yifan Zhou, Antti J Niemi

**Affiliations:** Department of Physics and Astronomy, Uppsala University, Uppsala, Sweden; Department of Biomedicine, Faculty of Medicine and Dentistry, University of Bergen, Jonas Lies Vei 91, NO-5009 Bergen, Norway; Laboratoire de Mathematiques et Physique Theorique CNRS UMR 6083, Fédération Denis Poisson, Université de Tours, Parc de Grandmont, F37200 Tours, France

**Keywords:** Side chain reconstruction, C_α_ trace problem, Rotamers, Protein visualisation

## Abstract

**Background:**

A commonly recurring problem in structural protein studies, is the determination of all heavy atom positions from the knowledge of the central α-carbon coordinates.

**Results:**

We employ advances in virtual reality to address the problem. The outcome is a 3D visualisation based technique where all the heavy backbone and side chain atoms are treated on equal footing, in terms of the C_α_ coordinates. Each heavy atom is visualised on the surfaces of a different two-sphere, that is centered at another heavy backbone and side chain atoms. In particular, the rotamers are visible as clusters, that display a clear and strong dependence on the underlying backbone secondary structure.

**Conclusions:**

We demonstrate that there is a clear interdependence between rotameric states and secondary structure. Our method easily detects those atoms in a crystallographic protein structure which are either outliers or have been likely misplaced, possibly due to radiation damage. Our approach forms a basis for the development of a new generation, visualization based side chain construction, validation and refinement tools. The heavy atom positions are identified in a manner which accounts for the secondary structure environment, leading to improved accuracy.

**Electronic supplementary material:**

The online version of this article (doi:10.1186/s12900-014-0027-8) contains supplementary material, which is available to authorized users.

## Background

Protein structure validation methods like MolProbity [1] and Procheck [2] help crystallographers to find and fix potential problems that are incurred during fitting and refinement. These methods are commonly based on *a priori* chemical knowledge and utilise various well tested and broadly accepted stereochemical paradigms. Likewise, template based structure prediction and analysis packages [3] and molecular dynamics force fields [4] are customarily built on such paradigms. Among these, the Ramachandran map [5,6] has a central role. It is widely deployed both to various analyses of the protein structures, and as a tool in protein visualisation. The Ramachandran map describes the statistical distribution of the two dihedral angles φ and ψ that are adjacent to the C_α_ carbons along the protein backbone. A comparison between the observed values of the individual dihedrals in a given protein with the statistical distribution of the Ramachandran map is an appraised method to validate the backbone geometry.

In the case of side chain atoms, visual analysis methods like the Ramachandran map have been introduced. For example, the Janin map [7] can be used to compare observed side chain dihedrals such as χ_1_ and χ_2_ in a given protein, against their statistical distribution, in a manner which is analogous to the Ramachandran map.

Crystallographic refinement and validation programs like Phenix [8], Refmac [9] and others, often utilize the statistical data obtained from the Engh and Huber library [10,11]. This library is built using small molecular structures that have been determined with a very high resolution. At the level of entire proteins, side chain restraints are commonly derived from analysis of high resolution crystallographic structures [12,13] in Protein Data Bank (PDB) [14]. A backbone independent rotamer library [15] makes no reference to backbone conformation. But the possibility that the side-chain rotamer population depends on the local protein backbone conformation, was considered already by Chandrasekaran and Ramachandran [16]. Subsequently both secondary structure dependent [17], see also [7] and [15], and backbone dependent rotamer libraries [18,19] have been developed. We note that the subject remains under active investigation [20-25].

The information content in the secondary structure dependent libraries and the backbone independent libraries essentially coincide [13]. Both kinds of libraries are used extensively during crystallographic protein structure model building and refinement. But for the prediction of side-chain conformations, for example in the case of homology modeling and protein design, there can be an advantage to use the more revealing backbone dependent rotamer libraries.

In x-ray crystallographical protein structure experiments, the skeletonisation of the electron density map is a common technique to interpret the data and to build the initial model [26]. The C_α_ atoms are located at the branch points between the backbone and the side chain. As such they are subject to relatively stringent stereochemical constraints; this is the reason why model building often starts with the initial identification of the skeletal C_α_ trace. The central role of the C_α_ atoms is widely exploited in structural classification schemes such as CATH [27] and SCOP [28], in various threading modeling techniques such as I-Tasser [29] and homology base approaches including SWISS-MODEL [30] and other related methods [31], in *de novo* approaches [32], and in the development of coarse grained energy functions for folding prediction [33]. As a consequence the so-called C_α_-trace problem has become the subject of extensive investigations [34-38]. The resolution of the problem would consist of an accurate main chain and/or all-atom model of the folded protein, based on the knowledge of the positions of the central C_α_ atoms only. Both knowledge-based approaches such and MAXSPROUT [34] and *de novo* methods including PULCHRA [37] and REMO [38] have been developed, to try and resolve the C_α_- trace problem. In the case of the backbone atoms, the geometric algorithm introduced by Purisima and Scheraga [39], or some variant thereof, is commonly utilized in these approaches. For the side chain atoms, most approaches to the C_α_ trace problem rely either on a statistical or on a conformer rotamer library in combination with steric constraints, complemented by an analysis which is based on diverse scoring functions. For the final fine-tuning of the model, all-atom molecular dynamics simulations can also be utilised.

In the present article we introduce and develop new generation visualisation techniques that we hope will become a beneficial complement to existing methods for protein structure analysis, refinement and validation. We use the C_α_ Frenet frames [40,41] to visualise the side chain. The output we aim at, is a 3D “what-you-see-is-what-you-have” type visual map of the statistically preferred all-atom model, calculable in terms of the C_α_ coordinates. As such, our approach should have value for example during the construction and validation of the initial backbone and all-atom models of a crystallographic protein structure.

Our approach is based on developments in three dimensional visualisation and virtual reality, that have taken place after the Ramachandran map was introduced. In lieu of the backbone dihedral angles that appear as coordinates in the Ramachandran map and correspond to a toroidal topology, we employ the geometry of virtual spheres that surround each heavy atom. We visually describe all the higher level heavy backbone and side chain atoms on the surface of a sphere, level-by-level along the backbone and side chains, exactly in the manner how they are seen by an imaginary, geometrically determined and C_α_ based miniature observer who roller-coasts along the backbone and climbs up the side chains, proceeding from one C_α_ atom to the next. At the location of each C_α_ our virtual observer orients herself consistently according to the purely geometrically determined C_α_ based discrete Frenet frames [40,41]. Thus the visualisation depends only on the C_α_ coordinates, and there is no reference to the other atoms in the initialisation of the construction. The other atoms - including subsequent C_α_ atoms along the backbone chain - are all mapped on the surface of a sphere that surrounds the observer, as if these atoms were stars in the sky.

At each C_α_ atom, the construction proceeds along the ensuing side chain, until the position of all heavy atoms have been determined. As such our maps provide a purely geometric and equitable, direct visual information on the statistically expected all- atom structure in a given protein.

The method we describe in this article, can form a basis for the future development of a novel approach to the C_α_ trace problem. As a complement to the existing approaches such as MAXSPROUT [34], PULCHRA [37] and REMO [38], the method we envision accounts for the secondary structure dependence in the heavy atom positions, which we here reveal. A secondary-structure dependent method to resolve the C_α_ trace problem should lead to an improved accuracy in the heavy atom positions, in terms of the C_α_ coordinates. In particular, since rotameric states do display clear secondary structure dependence, a fact that is sometimes overlooked in the development of rotamer libraries. The present article serves as a proof-of-concept.

## Method and results

### C_α_ based frenet frames

**Figure 1:**
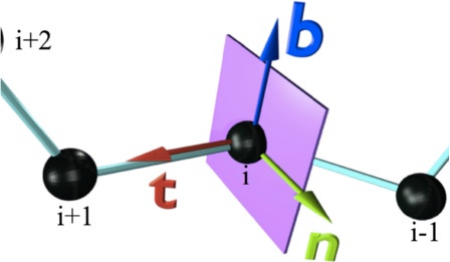
**Discrete Frenet frame.** (Color online) Discrete frenet frame vectors (1), (2) and (3).

Let **r**_i_ (*i* = 1,…, N) be the coordinates of the C_α_ atoms. The counting starts from the N terminus. At each **r**_i_ we introduce the orthonormal, right-handed, discrete Frenet frame (**t**_i_, **n**_i_, **b**_i_) [40]. As shown in Figure 1 the tangent vector **t** points from the center of the *i*^th^ central carbon towards the center of the (*i* + 1)^st^ central carbon,

1 $$ {\mathbf{t}}_i\kern0.5em =\kern0.5em \frac{{\mathbf{r}}_{i+1}-{\mathbf{r}}_i}{\left|{\mathbf{r}}_{i+1}-\left.{\mathbf{r}}_i\right|\right.} $$

The binormal vector is

2 $$ {\mathbf{b}}_i\kern0.5em =\kern0.5em \frac{{\mathbf{t}}_{i-1}\times {\mathbf{t}}_i}{\left|{\mathbf{t}}_{i-1}\times \left.{\mathbf{t}}_i\right|\right.} $$

The normal vector is

3 $$ {\mathbf{n}}_i\kern0.5em =\kern0.5em {\mathbf{b}}_i\times {\mathbf{t}}_i $$

We also introduce the virtual C_α_ backbone bond (*κ*) and torsion (*τ*) angles, as follows (see in Additional file 1: Figure S1),

4 $$ \cos {\kappa}_{i+1}\kern0.5em =\kern0.5em {\mathbf{t}}_{i+1}\cdot {\mathbf{t}}_i $$

5 $$ \cos {\tau}_{i+1}\kern0.5em =\kern0.5em {\mathbf{b}}_{i+1}\cdot {\mathbf{b}}_i $$

We identify the bond angle *κ* ∈ [0, π] with the latitude angle of a sphere which is centered at the C_α_ carbon. We orient the sphere so that the north-pole where *κ* = 0 is in the direction of **t**. The torsion angle τ ∈ [−π, π] is the longitudinal angle. It is defined so that τ = 0 on the great circle that passes both through the north-pole and through the tip of the normal vector **n**. The longitude angle increases towards the counter-clockwise direction around the vector **t**. Additional visual gain can be obtained, by stereographic projection of the sphere onto the plane. The standard stereographic projection from the south-pole of the sphere to the plane with coordinates (*x, y*) is given by

6 $$ x+iy\kern0.5em \equiv \kern0.5em \sqrt{x^2+{y}^2}{e}^{i\tau}\kern0.5em =\kern0.5em  \tan \left(\kappa /2\right){e}^{i\tau } $$

**Figure 2:**
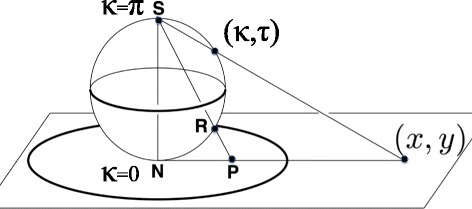
**Stereographic projection.** Standard stereographic projection of two-sphere on the plane, from the south- pole.

This maps the north-pole where *κ* = 0 to the origin (*x, y*) = (0, 0). The south-pole where *κ* = π is sent to infinity; see Figure 2. The visual effects can be further enhanced by sending

7 $$ \kappa \to f\left(\kappa \right) $$

where *f*(*κ*) is a properly chosen function of the latitude angle *κ*. Various different choices of *f*(*κ*) will be considered in the sequel.

### The C_α_ map

We first describe, how to visually characterize the C_α_ trace in terms of the C_α_ based Frenet frames (1)-(3). We introduce the concept of a virtual miniature observer who roller-coasts the backbone by moving between the C_α_ atoms. At the location of each C_α_ the observer has an orientation that is determined by the Frenet frames (1)-(3). The base of the *i*^th^ tangent vector **t**_i_ is at the position **r**_i_. The tip of **t**_i_ is a point on the surface of the sphere (*κ*, *τ*) that surrounds the observer; it points towards the north-pole. The vectors **n**_i_ and **b**_i_ determine the orientation of the sphere, these vectors define a frame on the normal plane to the backbone trajectory, as shown in Figure 1. The observer uses the sphere to construct a map of the various atoms in the protein chain. She identifies them as points on the surface of the sphere that surrounds her, as if the atoms were stars in the sky.

The observer constructs the C_α_ backbone map as follows [41]. She first translates the center of the sphere from the location of the *i*^th^ C_α_, all the way to the location of the (*i* + 1)^st^ C_α_, without introducing any rotation of the sphere, with respect to the *i*^th^ Frenet frames. She then identifies the direction of **t**_i+1_, i.e. the direction towards the site **r**_i+2_ to which she proceeds from the next C_α_ carbon, as a point on the surface of the sphere. This determines the corresponding coordinates (*κ*_i_, *τ*_i_). After this, she redefines her orientation to match the Frenet framing at the (*i* + 1)^st^ central carbon, and proceeds in the same manner. The ensuing map, over the entire backbone, gives an instruction to the observer at each point **r**_i_, how to turn at site **r**_i+1_, to reach the (*i* + 2)^nd^ C_α_ carbon at the point **r**_i+2_.

**Figure 3:**
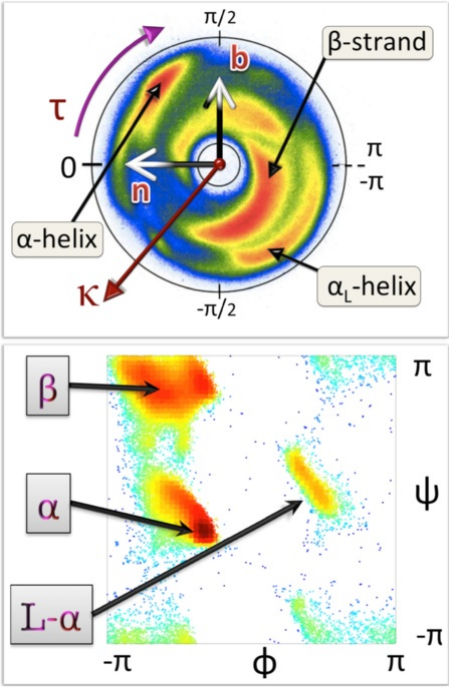
**C**_**α**_ **stereographical projection map and Ramachandran map.** (Color online) Top: The stereographically projected Frenet frame map of backbone C_α_ atoms, with major secondary structures identified. Also shown is the direction of the Frenet frame normal vector n; the vector t corresponds to the red circle at the center, and it points away from the viewer. The map is constructed using all PDB structures that have been measured with better than 2.0 Å resolution. Bottom: Standard Ramachandran map, constructed using our 1.0 Å resolution PDB subset. Major secondary structures have been identified.

In Figure 3 (top) we show the C_α_ Frenet frame backbone map. It describes the statistical distribution that we obtain when we plot all PDB structures which have been measured with better than 1.5 Å resolution, using the stereographic projection (6); in the sequel we then consider a subset with resolution better than 1.0 Å. There are presently 7548 entries measured with better than 1.5 Å resolution in PDB, and 488 entries with resolution better than 1.0 Å.

For our observer, who always fixes her gaze position towards the north-pole of the surrounding sphere at each C_α_*i.e.* towards the red dot at the center of the annulus, the color intensity in this map reveals the probability of the direction at position **r**_i_, where the observer will turn at next C_α_ carbon, when she moves from **r**_i+1_ to **r**_i+2_. In this way, the map is in a direct visual correspondence with the way how the Frenet frame observer perceives the backbone geometry. We note that the probability distribution concentrates within an annulus, roughly between the latitude angle values κ ~ 1 and κ ~ 3/2. The exterior of the annulus is a sterically excluded region while the entire interior is in principle sterically allowed but not occupied in the case of folded proteins. In the figure we identify four major secondary structure regions, according to the PDB classification. These are α-helices, β-strands, left-handed α-helices and loops. In this article we will use this rudimentary level PDB classification thorough.

We imagine surrounding C_α,i_ with sphere, with C_α,i_ at the origin, we may choose the radius of the sphere to coincide with the (average) virtual covalent bond length value which is 3.8 Å in the case of C_α_ atoms, excluding the *cis*-proline. See [42] for a recent statistical analysis of various virtual and non-virtual variables in protein structures. The variations in the covalent bond lengths are in general minor, and in this article we do not account for deviations in covalent bond lengths from their ideal values.

We note that the visualisation in Figure 3 (top) resembles the Newman projection of stereochemistry: The vector **t**_i_ which is denoted by the red dot at the center of the figure, points along the backbone from the promixal C_α_ at **r**_i_ towards the distal C_α_ at **r**_i+1_. This convention will be used thorough the present article.

For comparison, we also show in Figure 3 (bottom) the standard Ramachandran map. The sterically allowed and excluded regions are now intertwined, while the allowed regions are more localized than in Figure 3 (top). We point out that the map in Figure 3 (top) provides non-local information on the backbone geometry, it extends over several peptide units, and tells the miniature observer where the backbone turns at the next C_α_. As such it goes beyond the regime of the Ramachandran map, which is localized to a single C_α_ carbon and does not provide direct information how the backbone proceeds: The two Ramachandran angles φ and ψ are dihedrals for a given C_α_, around the N - C_α_ and C_α_ - C covalent bonds. These angles do not furnish information about neighboring peptide groups.

### Backbone heavy atoms

**Figure 4:**
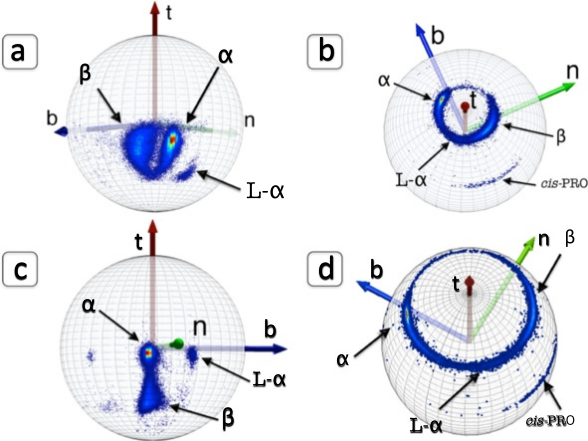
**Distributions of C**_**β**_ **, C, N, O atoms in Frenet frames.** (Color online) **a)** Distribution of C_β_ atoms in the C_α_ centered Frenet frames in PDB structures that have been measured with better than 1.0 Å resolution. The three major structures α-helices, β-strands and left-handed α-helices have been marked, following their identification in PDB. **b)** Same as **a)** but for backbone C atoms. Note that C atoms that precede a *cis*-proline are clearly identifiable. **c)** Same as **a)** and **b)** but for backbone N atoms. **d)** Same as **a)**, **b)** and **c)** but for backbone O atoms. As in **b)** the atoms preceding a *cis*-proline are clearly identifiable.

Consider our imaginary miniature observer, located at the position of a C_α_ atom and oriented according to the discrete Frenet frames. She observes and records the backbone heavy atoms N, C and the side-chain C_β_ that are covalently bonded to a given C_α_, and the O atom that is located in the peptide plane which is located after the given C_α_ along the backbone. In Figure 4a) - d) we show the ensuing density distributions, on the surface of the C_α_ centered sphere. These figures are constructed from all the PDB entries that have been measured using diffraction data with better than 1.0 Å resolution.

We note clear rotamer structures: The C_β_, C, N and O atoms are each localised, in a manner that depends on the underlying secondary structure [43]. Both in the case of C_β_ and N, the left-handed α-region (L-α) is a distinct rotamer which is detached from the rest. In the case of C and O, the L-α region is more connected with the other regions. But for C and O, the region for residues before *cis*-prolines becomes detached from the rest. In the case of C and C_β_ we do not observe any similar isolated and localised *cis*-proline rotamer.

The C and O rotamers concentrate on a circular region, with essentially constant latitude angle with respect to the Frenet frame tangent vector; for the O distribution, the latitude is larger. The N rotamers form a narrow strip in the longitudinal direction, while the map for C_β_ rotamers form a shape that resembles a horse shoe.

**Figure 5:**
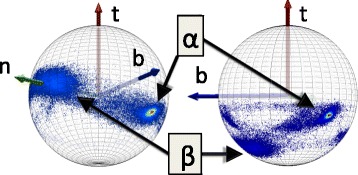
**Distributions of C**_**β**_ **, N atoms in REMO frame.** (Color online) Distributions of C_β_ atoms (left) and backbone N atoms (right) in the frames of REMO [36] are shown.

For comparison, in Figure 5 we visualise the C_β_ and N distributions in the coordinate system that is utilised in REMO [38]. In these frames, the secondary structures can be identified. But the rotamers are clearly much more delocalised than in the case of the Frenet frame map, shown in Figure 4a) and c). This delocalisation persists in the case of backbone C and O atoms (not shown). Similarly, we have found that in the case of the coordinate system of PULCHRA [37], the rotamers are similarly clearly more delocalised than in the Frenet frames (not shown).

One may argue that the stronger the localisation of rotamers, the more precise will structure analysis, prediction and validation become: Strong localisation enables a more precise identification of both outliers and misplaced atoms. From this perspective, the Frenet frames used here, appear to have a definite advantage over the frames used *e.g.* in PULCHRA and REMO.

Apparently, the secondary structure dependence of the distribution of the N, C and C_β_ atoms is mainly due to the Discrete Frenet Frame. However, we have to emphasize the secondary structures also deform the very local sp3-hybridized tetrahedron structure centered on C_α_ with the N, C and C_β_ atoms at corners. We consider the three bond angles

8 $$ {\vartheta}_{NC}\kern0.5em \equiv \kern0.5em N-{C}_a-C $$

9 $$ {\vartheta}_{N\beta}\kern0.5em \equiv \kern0.5em N-{C}_a-{C}_{\beta } $$

10 $$ {\vartheta}_{\beta C}\kern0.5em \equiv \kern0.5em {C}_{\beta }-{C}_a-C $$

**Figure 6:**
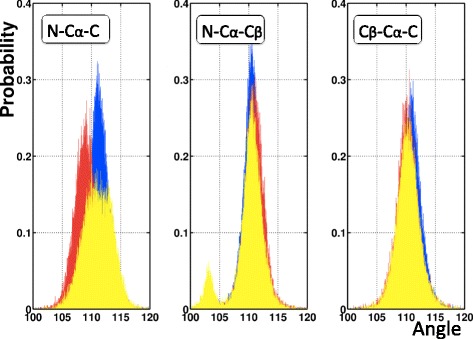
**Distribution of the three bond angles (** **8** **) - (** **10** **), according to secondary structures.** (Color online) Blue are α-helices, red are β-strands and yellow are loops; the small (yellow) peak in N-C_α_-C_β_ with angle around 103° is due to prolines. See Table 1 for the average values for α-helices, β-strands and loops in figure **a)**. See also Table 4 for the average values in figures **a)**, **b)** and **c)** with no regard to secondary structure. Finally, see Table 2 for the average values.

The *ϑ*_NC_ angle relates to the backbone only, while the definition of the other two involves the side chain C_β_. In Figure 6 we show the distribution of the three tetrahedral bond angles (8)-(10) in our PDB data set. We find that in the case of the two side chain C_β_ related angles *ϑ*_Nβ_ and *ϑ*_βC_, the distribution has a single peak which is compatible with ideal values; the isolated small peak in Figure 6b) is due to *cis*-prolines. But in the case of the backbone-only specific angle *ϑ*_NC_ we find that in our data set this is not the case. The PDB data set we use and display in Figure 6a) shows, that there is a correlation between the *ϑ*_NC_ distribution and the backbone secondary structure. See also Table 1.

**Table 1:** **Average values of the angle ϑ**_NC_ **for different secondary structures in figure** 6 **a)**

**Structure**	***ϑ***_**NC**_
Helix	111.5 ± 1.7
Strand	109.1 ± 2.0
Loop	111.0 ± 2.5

We note that in protein structure validation all three angles (8)-(10) are commonly presumed to assume the ideal values, shown in Table 2.

**Table 2:** **Average values of the angles in Figure** 6 **reported by various authors**

**Residue**	**EH-1**	**EH-2**	**AK**	**TV**
*ϑ*_NC_(PRO)		112.1 ± 2.6		112.8 ± 3.0
*ϑ*_NC_(REST)	110.5	111.0 ± 2.7	110.4 ± 3.3	111.0 ± 3.0
*ϑ*_Cβ_	110.1		110.1 ± 2.9	
*ϑ*_βN_	111.2		110.1 ± 2.8	

For example, the deviation of the C_β_ atom from its ideal position is among the validation criteria in MolProbity [1], that uses it to identify potential backbone distortions around C_α_. But several authors [43,44] have pointed out that certain variation in the values of the *ϑ*_NC_ can be expected, and is in fact present in PDB data. Accordingly, the protein backbone geometry does not appear to obey the single ideal value paradigm [10,11]; we refer to [15,18,19] for extended analysis.

We remind that *ϑ*_NC_ pertains to the two peptides planes that are connected by the C_α_. The Ramachandran angles (φ, ψ) are the adjacent dihedrals, but unlike *ϑ*_NC_ they are specific to a single peptide plane; the Ramachandran angles describe the twisting of the ensuing peptide plane. If the internal structure of the peptide planes is assumed to be rigid, the flexibility in the bond angle *ϑ*_NC_ remains the only coordinate that can contribute to the bending of the backbone. Consequently a systematic secondary structure dependence, as displayed in Figure 6, is to be expected. It could be that the lack of any observable secondary structure dependence in *ϑ*_Nβ_ and *ϑ*_βC_ suggests that existing validation methods distribute all refinement tension on *ϑ*_NC_.

### C_β_ atoms

The side chains are connected to the C_α_ backbone by the covalent bond between C_α_ and C_β_. Consequently the precision, and high level of localisation in the C_β_ map as shown in Figure 4a) becomes pivotal for the construction of accurate higher level side chain maps.

#### C_β_ at termini

We have analysed those C_β_ atoms that are located in the immediate proximity of the N and the C termini in the PDB data. For this, we have considered the first two C_β_ atoms starting from the N terminus, and the last two C_β_ atoms that are before the C terminus. Note that in the data that describes a crystallographic PDB structure, these do not need to correspond to the actual biological termini of the biological protein. In case the termini of the biological protein can not be crystallised, the PDB data describes the first two residues after the N terminus *resp.* the last two residues prior to the C terminus that can be crystallised. Here we consider the termini, as they appear in the PDB data.

**Figure 7:**
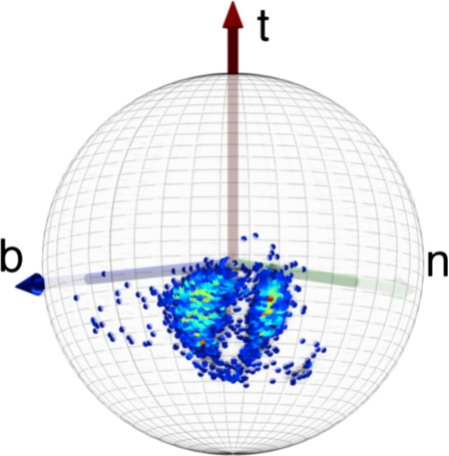
**Distribution of C**_**β**_ **atoms for terminal residues.** (Color online) The distribution of C_β_ directions in the first two and last two residues along PDB structures that have been measured using diffraction data with better than 1.0 Å resolution. There is no visible difference to the Figure 4a). In particular, there are very few clear outliers, and they are located mainly in the region left of the main region.

Recall, that the termini are commonly located on the surface of the protein. As such, they are accessible to solvent and quite often oppositely charged. It is frequently presumed that the termini are unstructured and highly flexible. They are normally not given any regular secondary structure assignment in PDB. But the Figure 7 shows that in the C_α_ Frenet frames the orientations of the two terminal C_β_ atoms are highly regular. Their positions on the surface of the C_α_ centered sphere are fully in line with that of all the other C_β_ atoms, as shown in Figure 4a). In particular, there are very few outliers. Moreover, the few outliers are (mainly) concentrated in a small region which is located towards the left from the β-stranded structures.

#### C_β_ and proline

Proline is different from the other amino acids, as its side chain connects to the backbone nitrogen atom N. There is an increased propensity to form *trans-*peptide planes. Thus, we analyze the distribution of proline and those amino acids that are its nearest neighbors separately, in detail.

**Figure 8:**
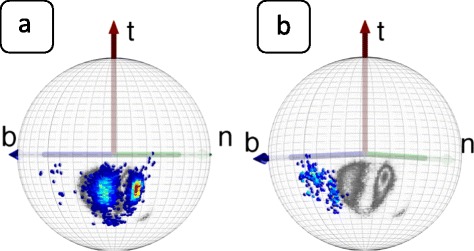
**The distribution of C**_**β**_ **in prolines.** (Color online) Figure **a)** is *trans*-PRO and figure **b)** is *cis*-PRO. The grey background is given by Figure 4a).

In Figure 8 we compare the individual proline contributions in our data set with the C_β_ background in Figure 4a). In Figure 8a) we show the *trans*-proline, and in Figure 8b) we show the *cis*-proline. The *trans*-proline has a very good match with the background. There are very few outliers. These outliers are predominantly located in the same region as in Figure 7, towards the left from the main distribution *i.e.* towards increasing longitude. We observe that all the *cis*-prolines are located outside of the main C_β_ distribution, towards the increasing longitude from the main distribution.

**Figure 9:**
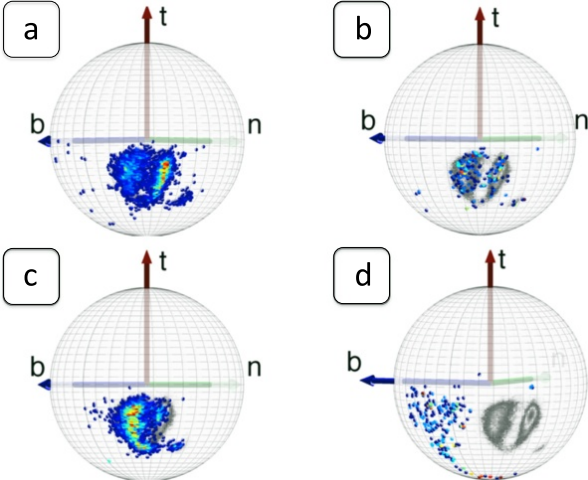
**Distribution of C**_**β**_ **atoms immediately after and right before a proline.** (Color online) The grey-scaled background is determined by the high-density region of Figure 4a). In figure **a)** immediately after *trans*-PRO and in figure **b)** immediately after *cis*-PRO. In figure **c)** right before *trans*-PRO and in figure **d)** right before *cis*-PRO.

In Figure 9a)-d) we display the C_β_ carbons that are located either *immediately after or right before* a proline. We observe the following:

In Figure 9a) we have the C_β_ that are immediately after the *trans*-proline. The distribution matches the background, with very few outliers that are located mostly in the same region as in Figures 7, 8 *i.e.* towards increasing longitude. But there is a *very* high density peak in the figure, that overlaps with the α-helical region: We remind that proline is commonly found right before the first residue in a helix.

In Figure 9b) we display those C_β_ atoms which are immediately after the *cis*- prolines. There is again a good match with the background. The *cis*-proline is relatively rare. Nevertheless, we observe an apparent increase in the number of points located in the β-stranded region. There are very few outliers, again mainly towards increasing longitude.

In Figure 9c) we have those C_β_ that are right before a *trans*-proline. There is a clear match with the background distribution. But there are relatively few entries in the α-helical position: It is known that helices rarely end in a proline. The intensity is very large in the loop region overlapping the β-strand region (see in Additional file 1: Figure S2); we always use the classification of the secondary structure of an entry, following PDB. There are also a few outliers. Again, the outliers are mainly located in the region towards increasing longitude.

In Figure 9d) we show the C_β_ distribution for residues that are right before a *cis*-proline. There are *no* entries in the background region of Figure 4a). The distribution is almost fully located in the previously observed outlier region, towards the left of the background in the figure. In addition, we observe an extension of this region towards increasing latitude, reaching all the way to the south-pole.

Finally, we demonstrate the effect of proline on the covalent tetrahedron which is centered on Cα atom. For this we recall that in Figure 4b) the region that corresponds to the effect of *cis*-prolines in the preceding C rotamer, is clearly visible. But in the case of C_β_ and N atoms, we do not observe any similar high density isolated *cis*-region.

**Figure 10:**
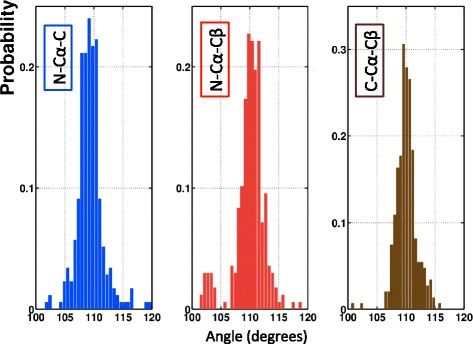
**Distribution of the three bond angles (** **8** **)-(** **10** **) in** ***cis*** **-proline.** (Color online) Distribution of the three heavy atom related angles (in degrees) in the C_α_ centered covalent tetrahedron, in the case of *cis*-proline. The numerical average values together with the one standard deviations are given in Table 3.

In Figure 10 we show the distribution of the three angles; see also Table 3. We observe a small deviation in the angle N - C_α_ - C. In comparison to proline values in Table 4, the value we find in our data set is smaller.

**Table 3:** **Average values of the angles in Figure** 10

**Angle**	***ϑ***_**NC**_	***ϑ***_**Cβ**_	***ϑ***_**βN**_
Average	109.3 ± 2.2	110.1 ± 1.8	110.0 ± 2.6

**Table 4:** **Average values of the angles in Figures** 6 **computed from our PDB data set**

**Angle**	***ϑ***_**NC**_	***ϑ***_**Cβ**_	***ϑ***_**βN**_
All	110.7 ± 2.3	110.5 ± 2.0	110.3 ± 2.4
PRO	112.6 ± 2.2	111.3 ± 1.7	103.2 ± 1.1
Rest	110.6 ± 2.3	110.4 ± 2.0	110.7 ± 1.7

The values are calculated without subdivision according to secondary structure. We also show the one-σ standard deviations. See also Table 2.

#### C_β_ and histidine

Histidine has a side chain with pKa around physiological PH. But we find that its C_β_ distribution is not affected by this property (see in Additional file 1: Figure S3).

### Level-γ rotamers

#### Standard rotamers

We proceed upwards along the side-chain, to the level-γ heavy atoms that are covalently bonded to C_β_. Conventionally, these atoms are described by the side-chain dihedral angle χ_1_. This angle is determined by the three covalently bonded heavy atoms C_α_, C_β_ and N. The angle χ_1_ determines the dihedral orientation of the level-γ carbon atom, in terms of these three atoms.

We remind that ALA and GLY do not contain any level-γ atoms. In the case of ILE and VAL we have two C_γ_ while in the case of CYS there is an S_γ_ atom.

We first define a χ_1_-framing, where the rotamer angle χ_1_ appears as a dihedral coordinate. For this we introduce the following C_α_ based orthonormal triplet

11 $$ {\mathbf{t}}_{x1}\kern0.5em =\kern0.5em \frac{{\mathbf{r}}_{\beta }-{\mathbf{r}}_a}{\left|{\mathbf{r}}_{\beta }-\left.{\mathbf{r}}_a\right|\right.} $$

12 $$ {\mathbf{n}}_{x1}\kern0.5em =\kern0.5em \frac{\mathbf{s}-{\mathrm{t}}_{x1}\left(\mathbf{s}\cdot {\mathbf{t}}_{x1}\right)}{\left|\mathbf{s}-{\mathrm{t}}_{x1}\left(\mathbf{s}\cdot {\mathbf{t}}_{x1}\right)\right|}\kern2em \mathrm{where}\mathbf{s}\kern0.5em =\kern0.5em {\mathbf{r}}_a-{\mathbf{r}}_{\beta } $$

13 $$ {\mathbf{b}}_{x1}\kern0.5em =\kern0.5em {\mathbf{t}}_{x1}\times {\mathbf{n}}_{x1} $$

with **r**_α_, **r**_β_ and **r**_N_ the coordinates of the pertinent C_α_, C_β_ and N atoms, respectively. This constitutes our χ_1_-framing, with C_α_ at the origin. We introduce a sphere around C_α_, oriented so that the north-pole is in the direction of **t**_*x*1_. Now the dihedral χ_1_ coincides with the ensuing longitude angle.

**Figure 11:**
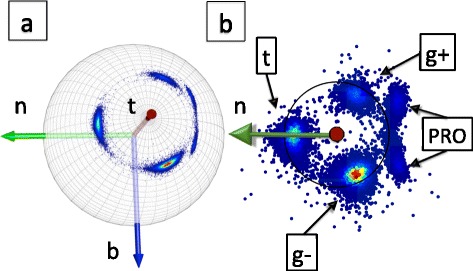
**C**_**γ**_ **atoms in the χ**_**1**_ **frames and its stereographic projection.** (Color online) **a)** C_γ_ atoms in the χ_1_-frames (11)- (13) on the C_α_ centered two-sphere. **b)** Stereographic projection of **a)** using (14). The three rotamers and proline are identified.

In Figure 11 we show the distribution of level-γ carbon atoms. The Figure 11a) shows the distribution on the surface of the C_α_ centered two-sphere. In Figure 11b) we use the stereographic projection (6) with the choice

14 $$ f\left(\kappa \right)\kern0.5em =\kern0.5em \frac{1}{1+ \exp \left\{{\kappa}^2\right\}} $$

in equation (7). The three rotamers *gauche* ± (g±) and *trans* (t) have been identified in this figure. The prolines are also visible, as rotamers. In addition, in Figure 11b) we have a circle that shows the average distance of the data points from the north-pole (origin) on the stereographic plane. A number of apparent outliers are visible in Figure 11b).

We note that the underlying secondary structure of the backbone is not visible in Figure 11. This is a difference between Figures 4 and 11, in the former the underlying backbone secondary structure is visible in the density profile.

**Figure 12:**
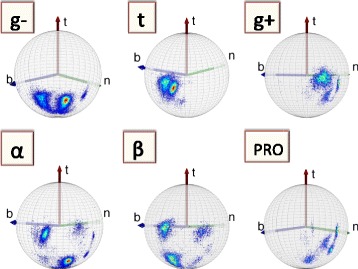
**Distribution of C**_**γ**_ **atoms in Frenet frame for different structures.** (Color online) Frenet frame view of the level-γ carbons, separately for the three rotamer states *g ±* and *t* (top line) and for α-helices, β-strands and prolines (bottom line).

In Figure 12 we show how the C_γ_ atoms are seen by the observer who is located at the C_α_ atom, and oriented according to the backbone Frenet frames; these are the frames used in Figure 4. Now both the rotamer structure and the various backbone secondary structures are clearly seen.

#### Secondary structure dependent level-γ rotamers:

In the C_α_ Frenet frame Figure 12 the secondary structure dependence is visible. But unlike Figure 11a) the C_α_ Frenet frame Figure 12 lacks an apparent symmetry. This complicates the implementation of the stereographic projection, such as the one shown in Figure 11b). We proceed to introduce a new set of frames, that enables us to analyse the secondary structure dependence of the γ-level atoms in terms of the stereographic projection:

We want this frame construction method to also remain valid for higher levels of side chains. For this we introduce the following notation. Suppose our observer is located at a generic atom X. She inquires about the distribution of another atom Y. She introduces an X centered frame as follows: With Z the atom where the observer made her previous observations, we set

15 $$ {\mathbf{t}}_X\kern0.5em =\kern0.5em \frac{{\mathbf{r}}_X-{\mathbf{r}}_Z}{\left|{\mathbf{r}}_X-{\mathbf{r}}_Z\right|} $$

16 $$ {\mathbf{n}}_X\kern0.5em =\kern0.5em \frac{{\mathbf{t}}_X\times {\mathbf{t}}_a}{\left|{\mathbf{t}}_X\times {\mathbf{t}}_a\right|} $$

17 $$ {\mathbf{b}}_X\kern0.5em =\kern0.5em {\mathbf{t}}_X\times {\mathbf{n}}_X $$

where **r**_Z_, **r**_X_ are the coordinates of atom Z and X and **t**_α_ is the tangent vector in the discrete Frenet frame.

In the case of C_γ_ level side chain, the atoms Z, X take C_α_ and C_β_, respectively. We may choose either C_α_ or C_β_ to coincide with the origin; the C_α_ centered coordinate system is the original roller coasting observer while the C_β_ centered coordinate system corresponds to an observer who has climbed “one-step-up” along the side chain. We map the level-γ atoms on the surface of the pertinent, surrounding two- spheres. We note that the difference between the C_α_ and C_β_ centered distributions appears mainly in the latitude i.e. in the distance from the north-pole (see in Additional file 1: Figure S4).

**Figure 13:**
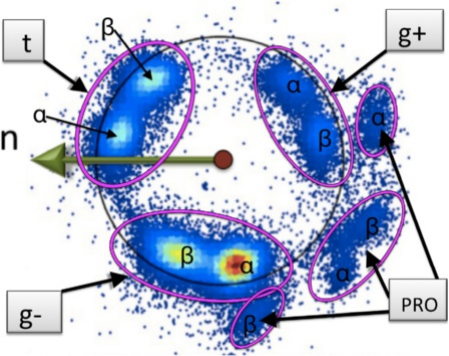
**Stereographic projection of level-γ rotamers in frames (** **15** **)-(** **17** **).** (Color online) Stereographic projection of level-γ rotamers in C_β_ frame in combination with (14) is shown.

In Figure 13 we have stereographically projected the distribution on the sphere, in combination with the map (14). The distribution displays clear localization, both in secondary structure and rotamer structure. The individual distributions for α-helices, β-strands and prolines are shown in Additional file 1: Figure S5, where a few outlying prolines are highlighted as examples. There are also outliers that are outside of the range of the stereographic projection in Figure 13. The projection - to the extent it has been plotted - covers a disk-like region around the north-pole i.e. around the tip of vector **t** in the figure. The far-away outliers can be visualised by properly rotating the sphere (see in Additional file 1: Figure S6).

Finally, we notice the fact that starting from γ-level atoms, the non-carbon heavy atoms appear in the side chain for some amino acids. However, it seems that these non-carbon heavy atoms obey the similar distributions as carbon atoms (see in Additional file 1: Figure S7).

### Level-δ rotamers

#### Standard dihedral angle

We proceed upwards along the side-chain, to describe level-δ atoms. We start with a coordinate frame which is centered at the C_γ_ atom. We note that in the case of ILE, two alternatives exist and we choose the C_γ_ carbon which is covalently bonded to the C_δ_ atom. We start with the standard way to describe the distribution of C_δ_ atom. It uses the dihedral angle χ_2_ defined in terms of the atoms C_α_, C_β_, C_γ_ and C_δ_. Correspondingly, a frame can be defined as

18 $$ {\mathbf{t}}_{x2}\kern0.5em =\kern0.5em \frac{{\mathbf{r}}_{\gamma }-{\mathbf{r}}_{\beta }}{\left|{\mathbf{r}}_{\gamma }-{\mathbf{r}}_{\beta}\right|} $$

19 $$ {\mathbf{n}}_{x2}=\kern0.5em \frac{{\mathbf{t}}_{x2}\times {\mathbf{t}}_a}{\left|{\mathbf{t}}_{x2}\times {\mathbf{t}}_a\right|} $$

20 $$ {\mathbf{b}}_{x2}\kern0.5em =\kern0.5em {\mathbf{t}}_{x2}\times {\mathbf{n}}_{x2} $$

**Figure 14:**
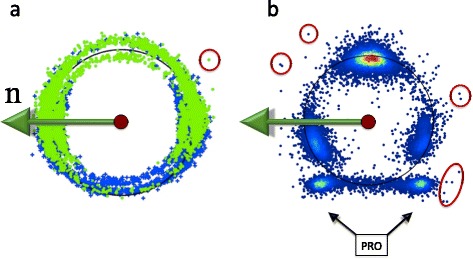
**Distribution of aromatic and non-aromatic level-δ C atoms in χ**_**2**_ **frames.** (Color online) **a)** Distribution of aromatic and **b)** non-aromatic level-δ C atoms, in the stereographic projection of the unit two-sphere centered at the C_γ_ atom. In **a)** the (dark) blue is C_δ1_ and (light) green is C_δ2_. Some outliers have been encircled, as examples. The (black) circles around the center denote the average distance of the distribution.

In Figure 14 we show the distribution of heavy atoms in level-δ, after stereographic projection (6). The longitude in these figures coincides with the standard χ_2_ dihedral angle, modulo a global π/2 rotation around the center. In addition, we introduce the following version of (7)

21 $$ f\left(\theta \right)\kern0.5em =\kern0.5em \frac{1}{1+{\theta}^4} $$

In Figure 14, we have separately displayed the distribution of the aromatic (a) and the non-aromatic (b) amino acids; we find that starting at level-δ this is a convenient bisection. We observe that the distributions in the case of aromatic and non-aromatic side chains are different. A clear trimodal rotamer structure is present in Figure 14b). Some outliers have been highlighted with circles, as generic examples. The individual distributions for PRO and O atoms at δ-level are shown in Additional file 1: Figure S8.

Finally, as in Figure 11 there is no visible sign of secondary structure in Figures 14: The standard χ_2_ dihedral is backbone independent.

**Figure 15:**
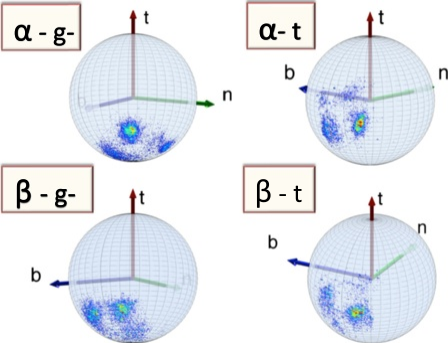
**Level-δ Frenet frame distributions corresponding to the level-γ distributions in Figure** 12 **.** (Color online) The labeling is as follows: *α-g −* stand for α-helical backbone secondary structure in *g −* rotamer in Figure 12, *α-t* stand for α-helical backbone secondary structure in *t* rotamer in Figure 12, *β-g −* stand for β-stranded backbone secondary structure in *g −* rotamer in Figure 12 and *β-t* stand for β-stranded backbone secondary structure in *t* rotamer in Figure 12.

However, as in Figure 12, in the backbone Frenet frames where the Cα is located at the center of the sphere, the secondary structure dependence becomes visible in the level-δ rotamers. As an example, we show in Figure 15 how some of the regions in Figure 12 are seen on the surface of the ensuing C_α_ centered sphere, by the roller coasting observer. The examples we have displayed are the overlap of the α-helical structures with the *g* − rotamer (marked *α-g −* in the figure) and *t* rotamer (*α-t*), and the overlap of the β-stranded structures with the *g −* rotamer (*β-g−*) and *t* rotamer *(β-t*). A secondary structure dependent trimodal rotamer structure is clearly present, in each of the distributions.

#### Secondary structure dependent level-δ rotamer angles

Following (15)-(17) and Figure 13 we proceed to visually inspect secondary structure dependence in the level-δ rotamers. In equations (15)-(17), the Z, X now correspond to the C_β_ and C_γ_ atoms, respectively.

**Figure 16:**
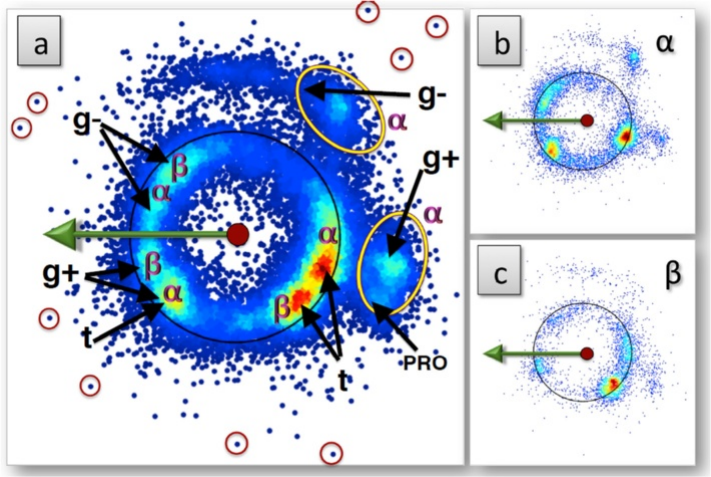
**The level-δ stereographical distribution of non-aromatic C atoms in frames (** **15** **)-(** **17** **).** (Color online) In figure **a)** we show the entire background, and in **b)** and **c)** those that have been classified as α-helical and β-stranded, respectively. Some outliers have also been marked.

We start with the non-aromatic amino acids. In Figure 16 we show the distribution of all the C_δ_ non-aromatic atoms in our data set. In this figure we have also identified those apparent rotamers that are classified either as α-helical or β-stranded in PDB. The figure shows that there is clear secondary structure dependence in these rotamers. The three corresponding level-γ rotamer subsets are also labeled in Figure 16a). To see it more clearly we draw the individual distributions for the three level-γ rotamer subsets and prolines (see in Additional file 1: Figure S9). Far-away outliers also exist (not shown), these can be located and visualised by rotating the original sphere as in Additional file 1: Figure S6. For the aromatic amino acids, we show all level-δ aromatic carbons (CD1 and CD2 in PDB) in Additional file 1: Figures S10 and S11. Again, the distributions of the secondary structures are localized well.

### Levels ε, ζ and η

Finally, we proceed to the ε, ζ and η levels. Following the analysis of C_γ_ and C_δ_ distributions, we use the frame (15)-(17) to visualise the secondary structure dependent distribution of these levels. For this, we introduce the analogous C_δ_, C_ε_ and C_ζ_ frames to display the distributions of atoms at the corresponding levels.

For the C_δ_ frame, we choose the atoms Z, X in equation (15)-(17) to coincide with the C_γ_ and C_δ_, respectively. Note that in the case of PHE and TYR two essentially identical choices can be made. In the case of TRP there are also two choices, and we choose the one denoted CD2 in PDB, it is covalently bonded to the higher level C atoms. In the case of HIS a framing could also be based on the level-δ N atom, but here we select the level-δ C atoms that are denoted CD2 in PDB.

**Figure 17:**
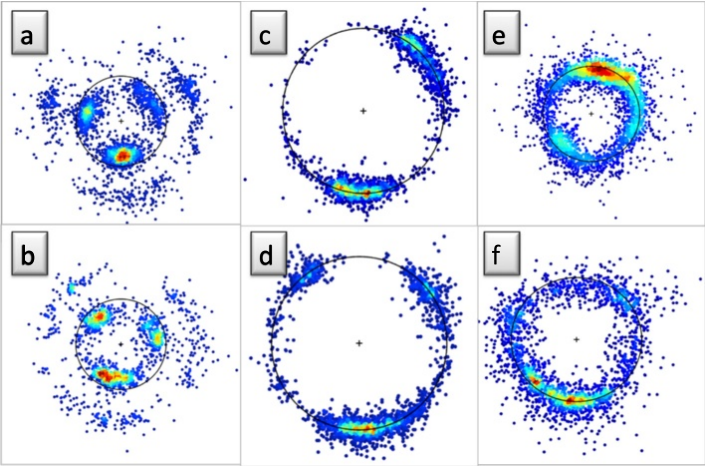
**Examples of rotamers in level-ε atoms.** (Color online) The black circles have the same radius in **a)** and **b)**, in **c)** and **d)**, and in **e)** and **f)**. In figure **a)** the α-helix and in **b)** the β-strand rotamers for for CE in MET and LYS; the structures outside the circle are LYS, those inside are MET. In figure **c)** the α-helix and in **d)** the β-strand rotamers for CE1 in PHE and TYR. In figure e) the α-helix rotamers for OE1 in GLU and GLN, and in f) the α-helix rotamers for OE2 in GLU (there is no GLN).

In Figure 17a) - f) we show various examples of level-ε atoms. We observe that in addition of rotamers in the longitude, there are also rotamer-like variations in the latitude angle, as shown in black circles in each figure.

**Figure 18:**
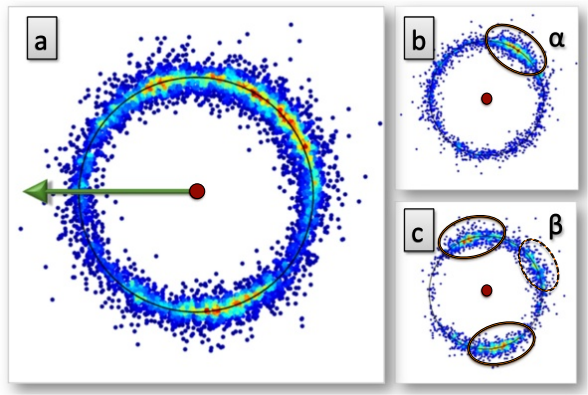
**Example of level-ζ rotamers.** (Color online) In figure **a)** we have all the C_ζ_ carbons in PHE and TYR. In figures **b)** and **c)** we show the subsets that correspond to α-helical and β-stranded secondary structures, respectively.

Similarly, we observe ζ-level atoms in C_ε_ frame, where Z and X atoms in equations (15)-(17) take atoms C_δ_ and C_ε_, respectively. As an example, in Figure 18 we identify one rotamer. In the case of β-stranded structures we observe three rotamers. We observe that the β-stranded rotamers are not distributed evenly. The rotamers are not related to each other by (regular) 120° rotations.

**Figure 19:**
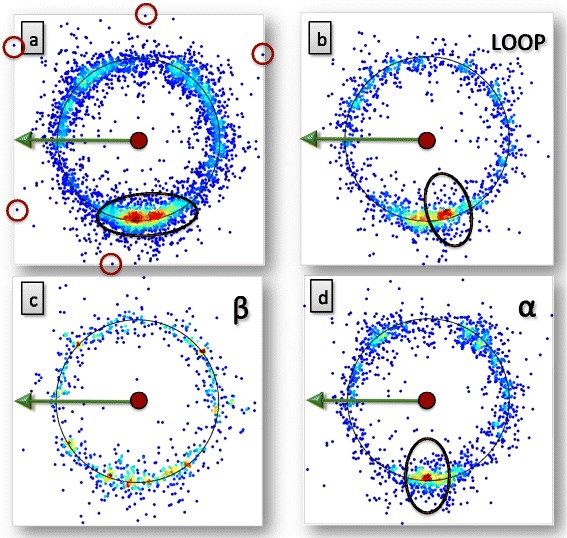
**Example of level-η rotamers.** (Color online) In figure **a)** we have all the Nη2 atoms in ARG. There are two very close rotamer states, which have been encircled. Some outliers have also been encircled. In figures **b)**-**d)** we show the subsets that correspond to loops, β-stranded and α-helical secondary structures, respectively. Comparison of the figures reveals that the two very close-by rotamers in **a)** correspond to loops and α-helices.

Finally, we use C_ζ_ frame to observe η-level atoms with Z and X taken as C_ε_ and C_ζ_ in the definition (15)-(17). As an example, the Nη2 distribution in ARG is shown in Figure 19. Now there is a very strong two-fold localisation of the distribution, shown in Figure 19a). Some of the outliers are encircled, as examples, in a).

## Discussion

We have utilised modern 3D visualisation techniques and advances in virtual reality to describe how to construct an entirely C_α_ geometry based visual library of the backbone and side chain atoms: There has been substantial progress in visualisation techniques, since the inception of the Ramachandran map. In lieu of a torus, our approach engages the geometry of a sphere and as such it has a direct “what-you- see-is-what-you-have” visual correspondence to the protein structure. In particular, we utilise the geometrically determined discrete Frenet frames of [40]. We propose the concept of an imaginary observer, chosen so that the discrete Frenet frames determine the orientation of the observer when she roller-coasts along the backbone and climbs up the side chains. She maps the directions of all the heavy atoms on the surface of a two-sphere that surrounds her, exactly as these atoms are seen in her local frame like stars in the sky.

Since the discrete Frenet frames can be unambiguously determined in terms of the C_α_ trace only, we can analyse both the backbone atoms and the side chain atoms on equal footing, in a single geometric framework. This is not possible in the conventional Ramachandran approach, that assumes *a priori* knowledge of the peptide planes, to define the dihedral angles.

As examples of the approach, we have analysed the orientation of various heavy atoms that are located both along the backbone and in the side chains. Our approach also enables a direct, *visual* identification of outliers.

In particular, we have found that in terms of the discrete Frenet frames, the secondary structure dependence becomes clearly visible in the rotamer structure, both in the case of the backbone atoms and in the case of the side chain atoms. Apparently this is not always the case, in conventional approaches such as [34,37,38]:

According to [13] conventional secondary structure dependent rotamer libraries do not provide much more information than backbone-independent rotamer libraries. But by using the Frenet frame coordinate system chosen here, we observe that there is a clear correlation between secondary structures and rotamer positions. Thus the approach we have presented, can form a basis for the future development of a novel approach to the C_α_ trace problem. As a complement to existing approaches [34,37,38] the one we envision accounts for the secondary structure dependence in the heavy atom positions that we have revealed, which should lead to an improved accuracy in determining the heavy atom positions.

## Conclusions

In this paper, we introduced a new method to visualise the heavy atom structure of a protein. In particular, our method easily detects those atoms in a crystallographic protein structure which are either outliers, or have been likely misplaced. Our approach can form a basis for the development of a new generation, visualisation based side chain construction, validation and refinement tool. Since the heavy atom positions are identified in a manner which correlates strongly with the secondary structure environment, this could lead to an improved accuracy, in particular when used in combination with existing methods.

## Additional file

Additional file 1:
**Some additional distribution examples for side-chain atoms.**

## References

[1] Chen VB, Arendall WB, Headd JJ, Keedv DA, Immormino RM, Kapral GJ, Murray LW, Richardson JS, Richardson DC (2010). MolProbity: all-atom structure validation for macromolecular crystallography. Acta Cryst D.

[2] Laskowski RA, MacArthur MW, Moss DS, Thornton JM (1993). PROCHECK**: a program to check the stereochemical quality of protein structures**. J App Cryst.

[3] Qu X, Swanson R, Day R, Tsai J (2009). A guide to template based structure prediction. Curr Protein Pept Sci.

[4] Freddolino PL, Harrison CB, Liu Y, Schulten Y (2010). Challenges in protein-folding simulations. Nature Phys.

[5] Ramachandran GN, Ramakrishnan C, Sasisekharan V (1963). Stereochemistry of polypeptide chain configurations. J. Mol. Biol..

[6] Carugo O, Carugo KD (2013). Half a century of Ramachandran plots. Acta Cryst D.

[7] Janin J, Wodak S, Levitt M, Maigret B (1978). Conformation of amino acid side-chains in proteins. J. Mol. Biol..

[8] Adams PD, Afonine PV, Bunkoczi G, Chen VB, Davis IW, Echols N, Headd JJ, Hung LW, Kapral GJ, Grosse-Kunstleve RW, McCoy AJ, Moriarty NW, Oeffner R, Read RJ, Richardson DC, Richardson JS, Terwilliger TC, Zwart PH (2010). PHENIX: a comprehensive Python-based system for macromolecular structure solution. Acta Cryst D.

[9] Murshudov GN, Vagin AA, Dodson EJ (1997). Refinement of macromolecular structures by the maximum-likelihood method. Acta Cryst. D..

[10] Engh RA, Huber R (1991). Accurate bond and angle parameters for X-ray protein structure refinement. Acta Cryst A.

[11] Engh RA, Huber R: **Structure quality and target parameters.** In: *International Tables for Crystallography. Vol. F.* Edited by Rossmann MG and Arnold E. Dordrecht, Kluwer Academic Publishers 2001: 382–392

[12] Ponder JW, Richards FM (1987). Tertiary templates for proteins: use of packing criteria in the enumeration of allowed sequences for different structural classes. J. Mol. Biol..

[13] Dunbrack RL (2002). Rotamer Libraries in the 21st Century. Curr. Op. Struc. Biol..

[14] Berman HM, Westbrookm J, Feng Z, Gilliland G, Bhat TH, Weissig H, Shindyalov IN, Bourne PE (2000). The protein data bank. Nucl. Acids Res..

[15] Lovell SC, Word J, Richardson JS, Richardson DC (2000). The penultimate rotamer library. Proteins.

[16] Chandrasekaran R, Ramachandran GN (1970). **Studies on the conformation of amino acids: XI. Analysis of the observed side group conformations in proteins**. Int J Protein Res.

[17] Schrauber H, Eisenhaber F, Argos P (1993). Rotamers: to be or not to be?: an analysis of amino acid side-chain conformations in globular. J Mol Biol.

[18] Dunbrack RL, Karplus M (1993). Backbone-dependent Rotamer library for proteins application to side-chain prediction. J. Mol. Biol..

[19] Shapovalov MS, Dunbrack RL (2011). A smoothed backbone-dependent Rotamer library for proteins derived from adaptive kernel density estimates and regressions. Structure.

[20] Islam SM, Stein R, Mchaourab H, Roux B (2013). Rotamer library of spin labeled cysteines attached to T4 lysozyme deduced from molecular dynamics simulations constrained by double electron–electron resonance (Deer) experiments. Biophys J.

[21] Alexander NS, Stein RA, Koteiche HA, Kaufmann KW, McHaourab HS, Meiler J (2013). RosettaEPR: rotamer library for spin label structure and dynamics. PloS One.

[22] Subramaniam S, Senes A (2012). **An energy-based conformer library for side chain optimization: improved prediction and adjustable sampling**. Proteins: Struct., Funct., Bioinf.

[23] Kirys T, Ruvinsky AM, Tuzikov AV, Vakser IA (2012). **Rotamer libraries and probabilities of transition between rotamers for the side chains in protein-protein binding**. Proteins: Struct, Funct, Bioinf.

[24] Subramaniam S, Senes A (2014). **Backbone dependency further improves side chain prediction efficiency in the Energy-based Conformer Library (bEBL)**. Proteins: Struct., Funct., Bioinf.

[25] Peterson LX, Kang X, Kihara D (2014). **Assessment of protein side-chain conformation prediction methods in different residue environments**. Proteins: Struct, Funct, Bioinf.

[26] Jones TA, Zou JY, Cowan SW, Kjeldgaard M (1991). Improved methods for building protein models in electron density maps and the location of errors in these models. Acta Cryst A.

[27] Sillitoe I, Cuff AL, Dessailly BH, Dawson NL, Furnham N, Lee D, Lees JG, Lewis TE, Studer RA, Rentzsch R, Yeats C, Thornton JM, Orengo CA (2013). New functional families (FunFams) in CATH to improve the mapping of conserved functional sites to 3D structures. Nucleic Acids Res.

[28] Murzin AG, Brenner SE, Hubbard T, Chothia C (1995). SCOP: A structural classification of proteins database for the investigation of sequences and structures. J. Mol. Biol..

[29] Roy A, Kucukural A, Zhang Y (2010). I-TASSER: a unified platform for automated protein structure and function prediction. Nature Protocols.

[30] Schwede T, Kopp J, Guex N, Peitsch MC (2003). SWISS-MODEL: an automated protein homology-modeling server. Nucleic Acids Res..

[31] Zhang Y (2009). Protein structure prediction: when is it useful?. Curr Opin Struct Biol.

[32] Dill K, Ozkan SB, Weikl TR, Chodera JD, Voelz VA (2007). The protein folding problem: when will it be solved?. Curr Op Struct Biol.

[33] Scheraga HA, Khalili M, Liwo A (2007). Protein-folding dynamics: overview of molecular simulation techniques. Ann Rev Phys Chem.

[34] Holm L, Sander C (1991). Database algorithm for generating protein backbone and side-chain coordinates from a Cα trace: Application to model building and detection of co-ordinate errors. Journ Mol Biol.

[35] DePristo MA, Bakker PIW, Shetty RP, Blundell TL (2003). Discrete restraint-based protein modeling and the Cα-trace problem. Prot. Sci..

[36] Lovell SC, Davis IW, Arendall WB, Bakker PIW, Word JM, Prisant MG, Richardson JS, Richardson DC (2003). Structure validation by Cα geometry: ψ, φ and Cβ deviation. Proteins.

[37] Rotkiewicz P, Skolnick J (2008). Fast procedure for reconstruction of full-atom protein models from reduced representations. Journ Comp Chem.

[38] Li Y, Zhang Y (2009). REMO: A new protocol to refine full atomic protein models from C-alpha traces by optimizing hydrogen-bonding networks. Proteins.

[39] Purisima EO, Scheraga HA (1984). Conversion from a virtual-bond chain to a complete polypeptide backbone chain. Biopolymers.

[40] Hu S, Lundgren M, Niemi AJ (2011). Discrete Frenet frame, inflection point solitons, and curve visualisation with applications to folded proteins. Phys. Rev. E.

[41] Lundgren M, Niemi AJ, Sha F (2012). Protein loops, solitons, and side-chain visualization with applications to the left-handed helix region. Phys Rev E.

[42] Hinsen K, Hu S, Kneller GR, Niemi AJ (2013). A comparison of reduced coordinate sets for describing protein structure. J Chem Phys.

[43] Lundgren M, Niemi AJ (2012). Correlation between protein secondary structure, backbone bond angles, and side-chain orientations. Phys Rev E.

[44] Touw WG, Vriend G (2010). On the complexity of Engh and Huber refinement restraints: the angle τ as example. Acta Cryst D.

