# Supplementary material for: A three dimensional visualisation approach to protein heavy-atom structure reconstruction
Source: BMC Struct Biol. 2014 Dec 31;14:27. doi: 10.1186/s12900-014-0027-8 (PMC4302604; doi:10.1186/s12900-014-0027-8)
Supplement: Additional file 1: — Some additional distribution examples for side-chain atoms. [file 12900_2014_27_MOESM1_ESM.pdf]

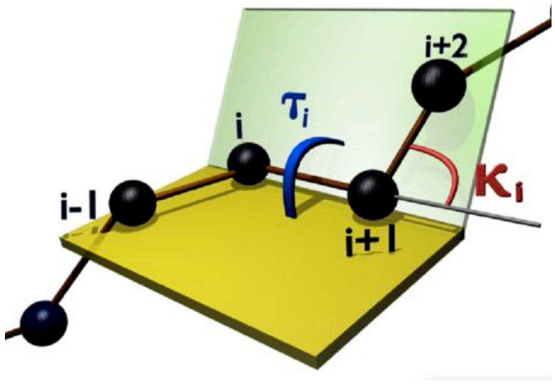

Figure 1 Bond and torsion angles (Color online) Geometry of bond (κ_i) and torsion (τ_i) angles.

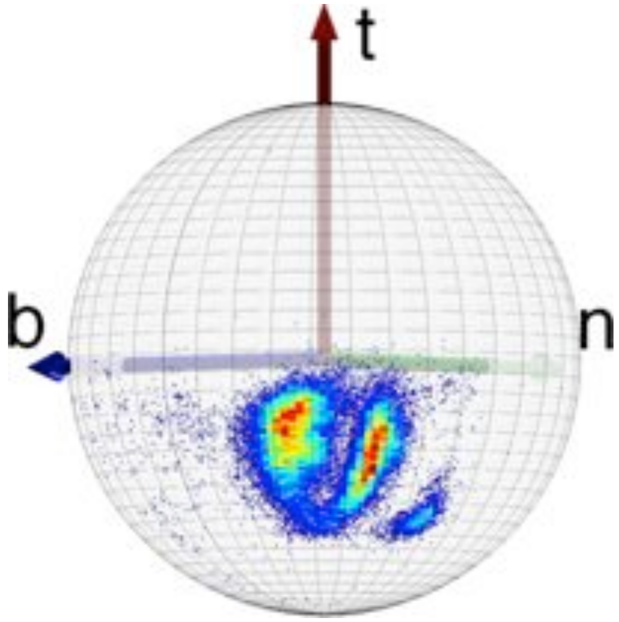

Figure 2 C$_\beta$ atom distribution for only loop part(Color online)From the figure, we can see that the C$_\beta$ atom distribution of loop part is severely overlapped with the secondary structure.

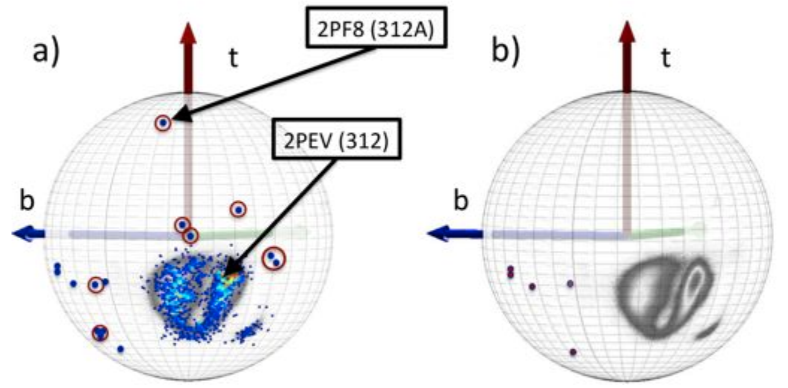

Figure 3 C_β distribution of histidine and histidine before *cis*-PRO (Color online) Figure a) shows C_β distribution of histidine. Some apparent outliers have been encircled, as examples. Residue number 312A in the PDB entry 2PF8 has been identified, together with 312 in the same protein 2PEV. Figure b) shows the subset of those HIS that precede a *cis*-PRO, there are five in our data set.

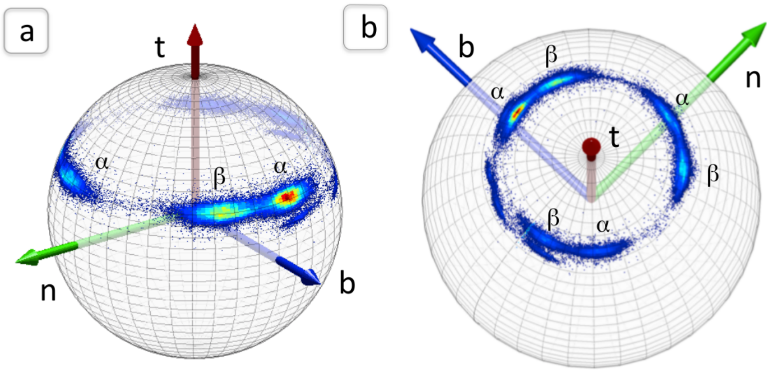

Figure 4 The level-γ atoms as seen in the coordinates Eq. (15)-(17) (Color online) In a) the origin coincides with the C_α atom, in b) it coincides with the C_β atom.

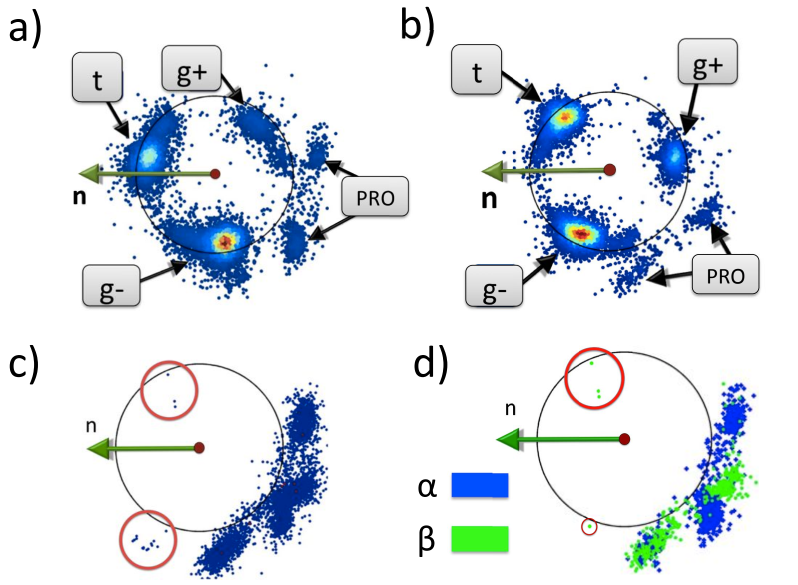

Figure 5 The identification of rotamers and major secondary structures of CG distribution in C_β frame (Color online) In a) the α-helices, in b) the β-strands. In c) all prolines, and in d) prolines divided according to their α-helical (blue) and β-stranded (green) assignment in PDB. Some apparent outliers have been high-lighted with red circles.

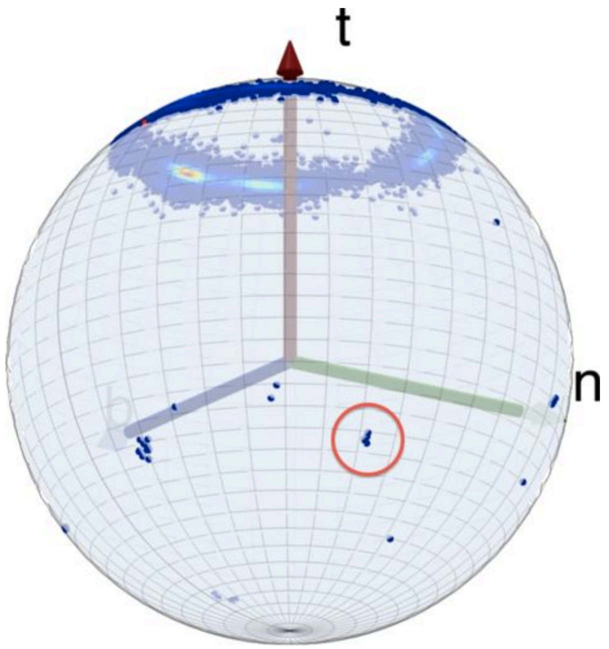

Figure 6 Outliers of C_γ atoms in C_β frame (Color online) An example of a group of five far away outliers in CG distribution, made visible by rotating the sphere and highlighting with red circle. They all correspond to the same protein but with different PDB codes: 1FN8, 1FY4, 1FY5, 1GDN and 1GDQ. In each case, the outlier is in the residue number 65 A. Note that there are also several other far away outliers.

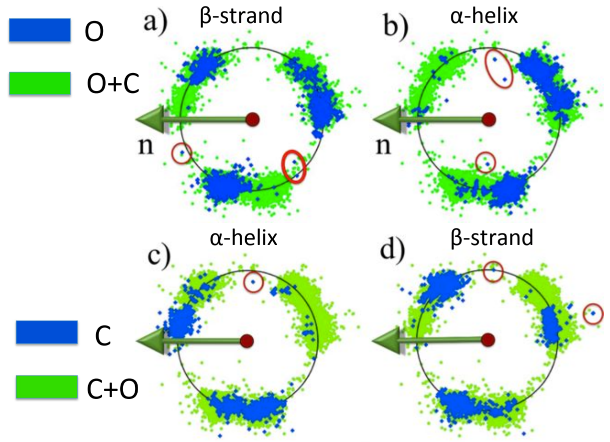

Figure 7 Distribution of O_γ and C_γ atoms of THR in different secondary structures (Color online) a) O_γ (dark blue) in THR, with backbone in the β-stranded position. b) O_γ (dark blue) in THR, with backbone in the α-helix position. c) Same as a) but for C_γ. d) Same as b) but for C_γ. Some apparent outliers are encircled. The (light green) background in each figure consists of all O_γ and C_γ in THR.

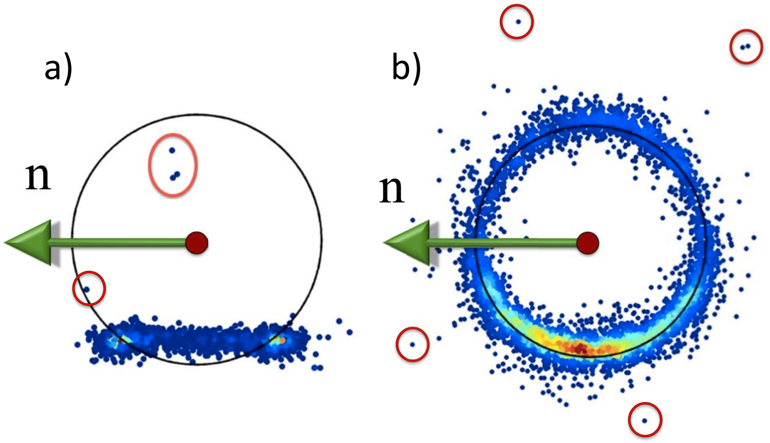

Figure 8 Distribution of level-δ C (only in PRO) and O atoms in $\mathcal{X}2$-frames (Color online) a) The proline contribution to the non-aromatic level-δ atoms distribution in $\mathcal{X}2$-frame. Three apparent outliers have been encircled. b) The level-δ distribution of O atoms. The (black) circle denotes the average distance of the distribution from the center. Some outliers have been highlighters with red circles.

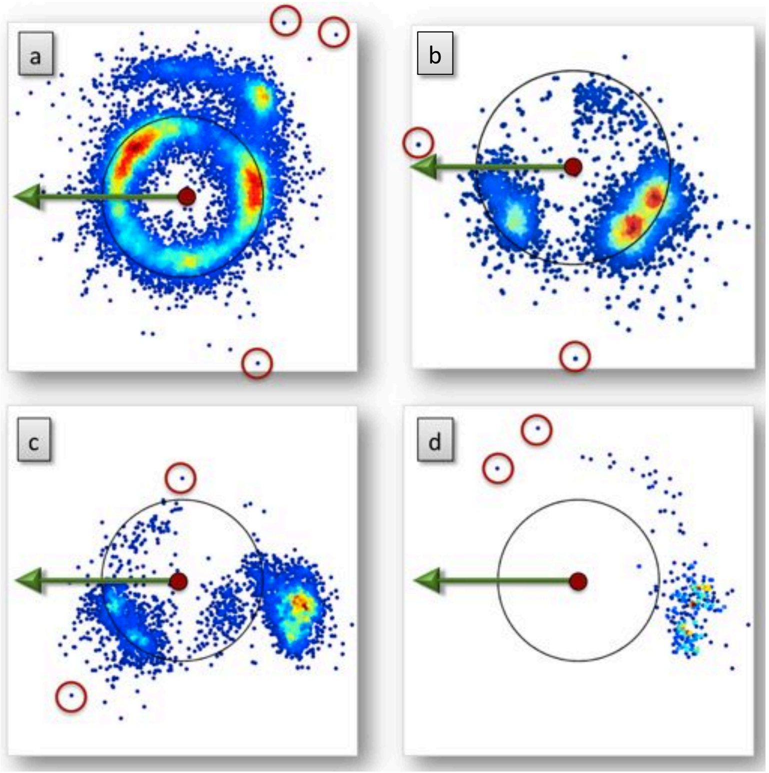

Figure 9 Identification of the regions of CD distributions according to the level-γ rotamers (Color online) The level-δ distribution of non-aromatic C atoms, in the stereographic projection and divided according to the level-γ rotamers. In figure a) we show the g- rotamer, in figure b) we show the t rotamer, in figure c) we have the $g+$ rotamer and in figure d) we show the cis-proline. The radii of the (black) circles coincide with the average latitude. Some outliers have also been encircled.

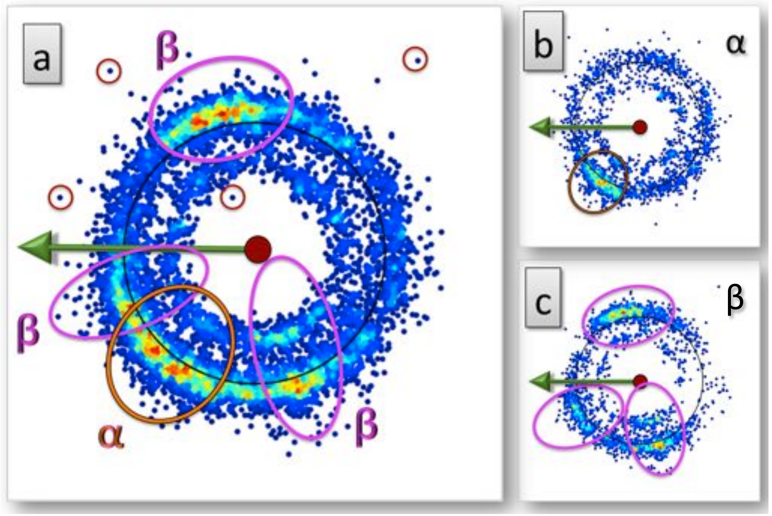

Figure 10 Distribution of aromatic CD1 atoms PHE, TYR and TRP in the stereographic projection (Color online) In a) all CD1 atoms in our data set, and in b) and c) the α-helical and β-stranded subsets, with rotamer states encircled . Some outliers have also been encircled in a).

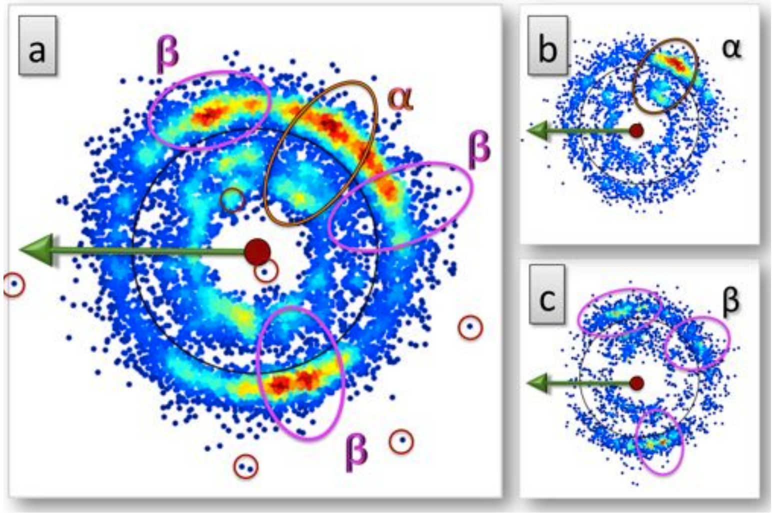

Figure 11 Distribution of aromatic CD2 atoms in stereographic projection (Color online) Same as figure 10, but for the CD2 carbons according to PDB classification, including PHE, TYR, TRP and HIS. Some outliers have also been encircled in subfigure a).
